# Reaction Pathways of Water Dimer Following Single Ionization

**DOI:** 10.1021/acs.jpca.3c07958

**Published:** 2024-02-26

**Authors:** Ivo S. Vinklárek, Hubertus Bromberger, Nidin Vadassery, Wuwei Jin, Jochen Küpper, Sebastian Trippel

**Affiliations:** † Center for Free-Electron Laser Science CFEL, Deutsches Elektronen-Synchrotron DESY, Notkestraße 85, 22607 Hamburg, Germany; ‡ Center for Ultrafast Imaging, Universität Hamburg, Luruper Chaussee 149, 22761 Hamburg, Germany; § Department of Physics, Universität Hamburg, Luruper Chaussee 149, 22761 Hamburg, Germany

## Abstract

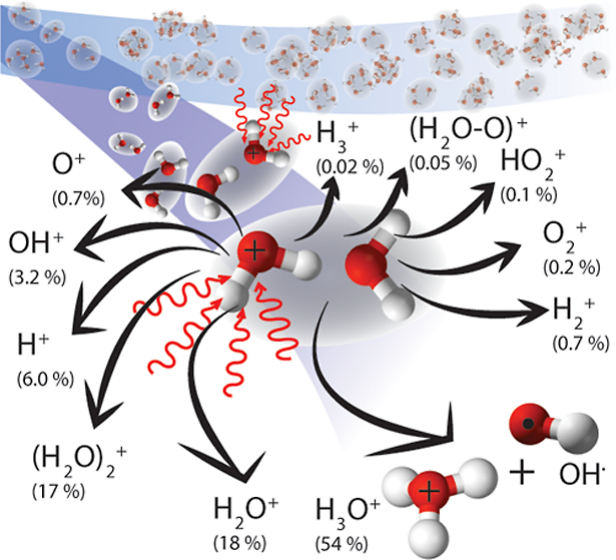

Water dimer (H_2_O)_2_—a vital component of the earth’s atmosphere—is an important prototypical hydrogen-bonded system. It provides direct insights into fundamental chemical and biochemical processes, e.g., proton transfer and ionic supramolecular dynamics, occurring in astro- and atmospheric chemistry. Exploiting a purified molecular beam of water dimer and multimass ion imaging, we report the simultaneous detection of all generated ion products of (H_2_O)_2_^+^ fragmentation following single ionization. Detailed information about ion yields and reaction energetics of 13 ion-radical pathways, 6 of which are new, of (H_2_O)_2_^+^ are presented, including strong ^18^O-isotope effects.

## Introduction

Water dimer (H_2_O)_2_ is assumed to be a vivid contributor to the radiation budget,^1,2^ the homogeneous condensation,^3^ and chemical reactions including degradation of Criegee intermediates^4^ at low altitudes of the earth’s atmosphere.^5−7^ Moreover, the decay of the ionic states of water clusters induced by cosmic radiation is considered essential for astrochemistry occurring on ice mantles^8−10^ and plays a key role as a trigger of chemical evolution in interstellar space.^11^ Therefore, as (H_2_O)_2_ is the smallest water cluster, it is a favorable model system to develop a more thorough understanding of the environmental effect, i.e., hydrogen bonding’s role, in energy, charge, and mass transfer occurring in ion-radical chemistry.^12−19^

Hydrogen bonding is of major importance for the vast majority of biochemical processes and chemical reactions such as proton transfer in redox reactions of light-harvesting complexes^20^ or structure and topological stability of biomolecules like the double-helix structure of DNA.^21,22^ Specifically, understanding how absorbed energy dissipates and charge is redistributed within an ionized aqueous environment is crucial in a biological context, where the interaction of biomolecules with generated low-energy electrons and ion radicals can result in their degradation.

The interest in ionic supramolecular dynamics of the (H_2_O)_2_ system led to several theoretical predictions^15,16,18,23^ of fragmentation pathways of singly and doubly charged water dimer and fragmentation energetics. These were only partly supported by the experimental results of electron-impact ionization^24^ and of Coulomb explosion^12,14,19,25,26^ induced by core and valence ionization. This is not surprising, considering the experimental challenge, e.g., the interference of the products of (H_2_O)_2_^+^ fragmentation with those resulting from the fragmentation of higher clusters and isolated H_2_O molecules. Our experimental approach circumvents these issues by using the electrostatic deflector^27^ for the purification of (H_2_O)_2_ samples and multimass ion imaging^28−30^ to monitor all the ion-radical channels induced by strong-field ionization at once. The photoionization was set to be predominantly in the multiphoton regime to populate similar states of the (H_2_O)_2_^+^ ion as those populated from VUV/UV- or electron-impact ionization.

Ab initio simulations revealed that the photoionization of (H_2_O)_2_ through electron ejection from the four highest occupied molecular orbitals (HOMO to HOMO – 3) preferably leads to proton transfer.^15^ The first two ionic states of (H_2_O)_2_^+^, i.e., the ground ^2^*A*″ and the first excited ^2^*A*′ states, are populated by electron ejection from the two nonbonding 1b_1_ orbitals of oxygen in the proton donor (HOMO) and the proton acceptor (HOMO – 1), respectively.^15,18^ Both of these states are highly reactive, which is underlined by the estimated time scale for proton migration of less than 100 and 300 fs for (H_2_O)_2_^+^ in its ^2^*A*″ and ^2^*A*′ states, respectively.^15^ Recently, the time scale for the proton migration was measured as 55(20) fs in XUV-pump-XUV-probe experiments.^26^ Subsequently, (H_2_O)_2_^+^ either dissociates into
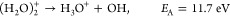
1or survives as an ion-radical pair (<40%)^15,16^
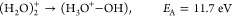
2*E*_A_ denotes the specific appearance energy, i.e., the minimum energy required to induce the reaction pathway.

The next two excited states of (H_2_O)_2_^+^ are populated through electron ejection from the 3a_1_ orbital of donor (HOMO–2) and acceptor (HOMO – 3) H_2_O followed by the prompt decay^15^ into the ^2^*A*″ and ^2^*A*′ states with subsequent dissociation via the channel in (1). Additional minor channels (<20%) either lead to an ion-radical pair (H_2_O)_2_^+^ through the channel in (2) or fragmentation without proton transfer as
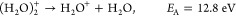
3

Furthermore, the electron ejection from the 1b_2_ orbital of the donor (HOMO–4) and acceptor (HOMO–5) predominantly leads to the three-body fragmentation as
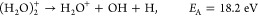
4generating four species: three radical fragments and an electron. Those species should eminently increase the possibility of radiation damage in an aqueous environment.^15^ The two-body fragmentation channels producing H_3_O^+^ and H_2_O^+^ ions participate only with a minor contribution (<20%) after HOMO–4 and HOMO–5 ionization.^15^

Additional channels of (H_2_O)_2_^+^ fragmentation appearing at higher ionization energies were described,^15,16^ i.e.
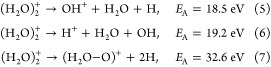
5

Contrary to valence ionization, stripping of a core electron from the 2a^1^ or 1a^1^ orbitals, e.g., by X-ray ionization, induces direct or proton-transfer-mediated^13,17^ relaxation through local or intermolecular Auger decay^12−14,17^ and subsequent ejection of a valence electron.

Here, we exploited the combination of electrostatic deflection^31^ and multimass imaging with a Timepix3 camera^28,29^ to yield unprecedented details into the ionic reaction pathways of the prototypical ionized (H_2_O)_2_ system. We produced a rotationally cold molecular beam of (H_2_O)_2_ with ∼92% purity^27^ and recorded all ionic reaction products, see Methods and the Supporting Information for details. We directly observed all theoretically predicted fragmentation channels of (H_2_O)_2_^+^.^15,16^ Moreover, we observed multiple new fragmentation channels. The velocity-map-imaging (VMI) detection inherently provided information about the released translational energy and rovibronic excitation of the products, which could affect subsequent reactions.

## Methods

**Figure 1:**
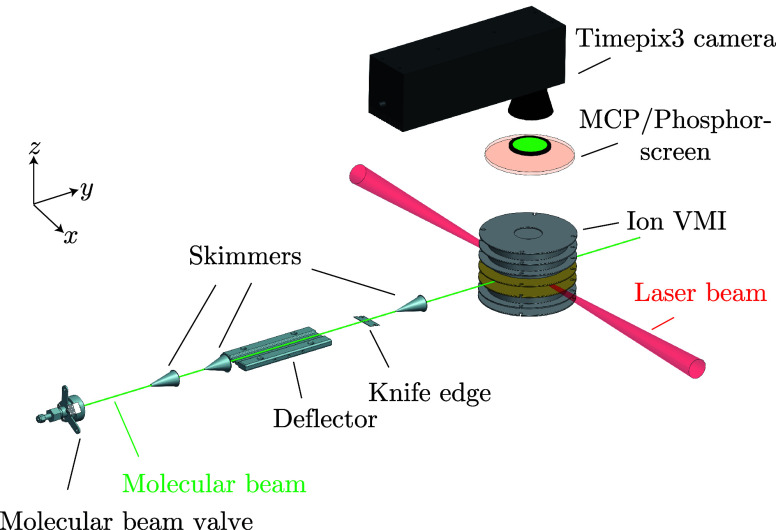
Schematic picture of the endstation for controlled-molecule experiments (eCOMO).^32,33^

All experiments were performed in our recently commissioned transportable endstation for controlled-molecule experiments (eCOMO), which will be described in more detail elsewhere.^32^Figure 1 provides a sketch of the experimental setup.^32,33^ A sample of distilled water (0.5 μL, room temperature) was dropped on a glass-filter paper and installed in a sample holder behind the pulsed valve (Amsterdam Piezo Valve).^34^ The water vapor was mixed with helium buffer gas using a stagnation pressure of 4 bar and the mixture coexpanded into vacuum in 50 μs pulses into the source chamber; pressures with the valve on/off were 2 × 10^–6^ mbar/2 × 10^–8^ mbar. The molecular beam of water clusters was extracted by a first skimmer (diameter of 3 mm) from the supersonic jet and further collimated by a second skimmer (diameter of 1.5 mm) before entering the electrostatic deflector.

To spatially separate the water dimer clusters from the carrier helium gas as well as from isolated water molecules and larger water clusters, we used a *b*-type deflector,^31,35^ applying a voltage of 13 kV. The dipole moments of water monomer and dimer are 1.86 and 2.63 D, respectively.^27,36,37^ Subsequently, the molecular beam was cut in half by a knife edge to increase both, the effective separation of water dimer from the rest of the molecular beam and the column density.^33^ The source, deflector, knife edge, and skimmers are all movable, which enabled us to optimize their positions for the separation and purification of the water-dimer clusters. The deflected molecules were intersected with short laser pulses in the interaction region of a double-sided VMI spectrometer. The purity of the water-dimer beam was estimated to be 92% by comparing the signals from known fragmentation channels to the background signal, i.e., water monomer, nitrogen, and oxygen, at the deflected-beam position of 2 mm.

To photoionize the molecules, we used an 800 nm Ti/sapphire chirped-pulse-amplifier system (Coherent Astrella) operated at 1 kHz. The pulse duration is estimated to be 40 fs and the pulse energies, 170 μJ. Focusing the laser beam to 53 μm full width at half-maximum intensity yielded a peak intensity up to ∼2 × 10^14^ W/cm^2^, corresponding to a Keldysh parameter of ∼0.7 for photoionization of (H_2_O)_2_ at *E*_i_ = 11.7 eV.^38^

The generated ions were then accelerated toward the detector in a perpendicular geometry of the double-sided spectrometer^39^ and projected under VMI conditions. At the end of the spectrometer, a microchannel-plate–phosphor-screen combination (MCP: Photonics, APD 3 PS 75/32/25/8 I 60:1 NR MGO 8″FM; PS: P47) was mounted to produce light flashes for individual ions. The flashes were detected with a Timepix3 camera^28,40^ (Amsterdam Scientific Instruments) operated and controlled by our open-source-library PymePix,^41,42^ which was also used to extract the raw physics events from the Timepix3 data stream. The detector’s temporal resolution of ∼1.6 ns^28^ enabled us to work in multimass-detection mode and to obtain the VMI images of all fragments directly by slicing, i.e., computational event selection, in the time-of-flight coordinate. The synchronization of the whole experiment is provided by a Stanford DG645 delay generator.

The plotted VMI images and beam-plot figures were background-subtracted and also stripped off the contribution from the scattered/nondeflected beam by subtraction of the measured background-subtracted signal with the deflector off. The valve was operated at 200 Hz resulting in 4 successive background measurements due to the 5 times higher repetition rate of the laser with respect to the molecular-beam valve. The data were centroided by calculating the center of mass for each detected ion.^28^ The 3D velocity (radial) and angular distributions were obtained by standard integration methods for VMI images.^43^ For momentum calibration,^28^ we fitted the dependence of the center positions of the VMI images on the time of arrival and converted it to momenta applying the physical size of the ion detector and the magnification factor (*M* = *v*_i_/*v*_mb_ = 0.843) of the spectrometer estimated by SIMION.^44^ The resulting velocity of the molecular beam is 2 km/s.

## Results and Discussion

**Figure 2:**
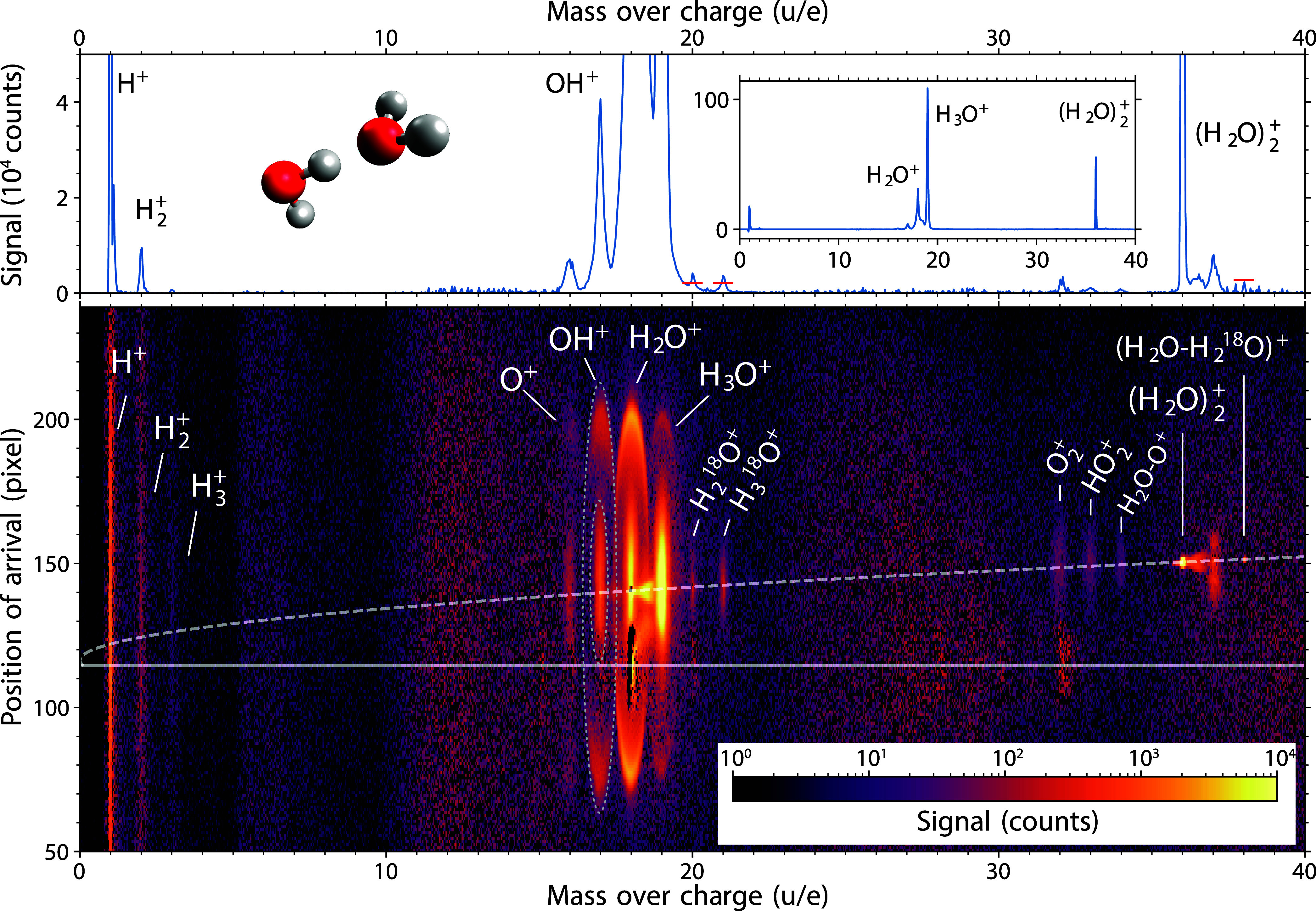
Image mapping the ion signal according to the mass-over-charge ratio and the position of arrival on the detector (lower panel). The upper panel outlines the zoomed-in mass spectrum, with an inset showing the whole vertical range for reference. The observed fragments of (H_2_O)_2_^+^ are H^+^, H_2_^+^, H_3_^+^, O^+^, OH^+^, H_2_O^+^, H_3_O^+^, O_2_^+^, HO_2_^+^, and (H_2_O–O)^+^. The observed fragments of (H_2_O–H_2_^18^O)^+^ are H_2_^18^O^+^ and H_3_^18^O^+^. The expected isotopologue-peak heights of H_2_^18^O^+^, H_3_^18^O^+^, and (H_2_O–H_2_^18^O)^+^ ions are indicated by the red horizontal bars. Dotted ellipses at *m*/*q* = 17 u/e are drawn to mark product regions of (H_2_O)_2_^+^ fragmentation and Coulomb explosion as an example. See text for details.

Figure 2 depicts an overview of all acquired ions and their fragmentation channels resulting from singly and multiple-charged water dimers after strong-field ionization at 800 nm. The image shows the background-subtracted ion signal for the mass-over-charge ratio and the position of arrival at the detector; see the Supporting Information for details. The mass-over-charge ratio *m*/*q* is obtained by a direct transformation from the ion time of flight by *m*/*q* ∝ *t*^2^. Due to the molecular beam velocity, in this two-dimensional spectrum, all signals from species originating from the molecular beam are shifted upward in the figure with increasing mass-over-charge ratio. Ions with zero velocity in the co-moving frame of the molecular beam appear on the dashed line. Ions in the surrounding structures centered at the dashed line are due to fragmentation of the cluster after ionization. The remaining signals are due to the ionization of the diffuse rest gas in the chamber.

For a direct comparison between the various fragments in terms of signal strength, the inset in the top graph of Figure 2 depicts the mass spectrum obtained by the summation of the signal in the lower figure along the vertical axis. The top figure itself is a zoom into the inset to highlight weak channels.

The most prominent features in Figure 2 are observed in the region between *m*/*q* = 16 and 19 u/e, corresponding to the O^+^, OH^+^, H_2_O^+^, and H_3_O^+^ fragments. These fragments originated from the (H_2_O)_2_^+^ fragmentation and Coulomb-explosion channels of (H_2_O)_2_^2+^. We were able to distinguish between these two sources of the signal using the 3D ion velocity detection with the Timepix3 camera.^28^ Whereas (H_2_O)_2_^+^ fragmentation is represented by the central broad features around the dashed line, e.g., confined by a inner dotted ellipse at *m*/*q* = 17∼u/e, the Coulomb-explosion channels from (H_2_O)_2_^2+^ exhibit sharp ring-like—graphically projected, oval—structures assigned to “fast” ions, e.g., the area between the two dotted ellipses around *m*/*q* = 17∼u/e, which will be discussed in a future publication. The strong peaks at *m*/*q* = 16 to 19 u/e are also accompanied by weak signals at *m*/*q* = 20 and 21 u/e assigned to the isotopologues H_2_^18^O^+^ and H_3_^18^O^+^, respectively. The signals corresponding to the expected peak heights for isotopes in natural abundance, i.e., 0.2% of the ^16^O-isotopologue signal, are indicated by the red horizontal lines in the mass spectrum of Figure 2. The red bar assigned to H_2_^18^O^+^ was corrected upward to compensate for the contribution from the neighboring H_3_O^+^ signal.

The second region with a strong signal, at *m*/*q* = 36 u/e, corresponds to the parent ion (H_2_O)_2_^+^. Moreover, it is accompanied by a weak peak of its isotopologue (H_2_O–H_2_^18^O)^+^ at *m*/*q* = 38 u/e, again with its expected natural-abundance signal contribution indicated by a red line in the mass spectrum. The signal strength is in very good agreement with the expected abundance. The well-resolved and clear observation of these isotopologues demonstrates the high sensitivity of our experiment. In between the two peaks, there is another structure at *m*/*q* = 37 u/e, which we assign to protonated water dimer (H_2_O)_2_H^+^ originating from the fragmentation of larger clusters (H_2_O)_*n*_^+^, *n* > 2, i.e., a remaining impurity in the experiment.

For *m*/*q* ratios of 32, 33, and 34 u/e, we also observed weak but distinct signals originating in the molecular beam. These signals were assigned to O_2_^+^, HO_2_^+^, and (H_2_O–O)^+^ ions,^16^ respectively. The origin of these fragments is not straightforwardly linked to any specific ion precursor. Nevertheless, measurements without purification of the reactant by deflection do not exhibit any significantly intense signals between *m*/*q* = 32 and 34 u/e, see Figure S1 in the Supporting Information, which excludes their origin from larger water clusters (H_2_O)_*n*_ or mixed (H_2_O)_*m*_(O_2_)_*n*_ clusters. Furthermore, there are no coincidences of the O_2_^+^, HO_2_^+^, and (H_2_O–O)^+^ ions with H^+^ and H_2_^+^ ions, see Figure S2 in the Supporting Information. Therefore, we assign the signal at 32, 33, and 34 u/e to fragmentation products of (H_2_O)_2_^+^.

The last region with a distinguishable signal is located at *m*/*q* = 1–3 u/e. We assign the signal to the hydrogen ions H^+^, H_2_^+^, and H_3_^+^, as the isotopic natural-abundance contribution of deuterium D is below 0.02%.

**Figure 3:**
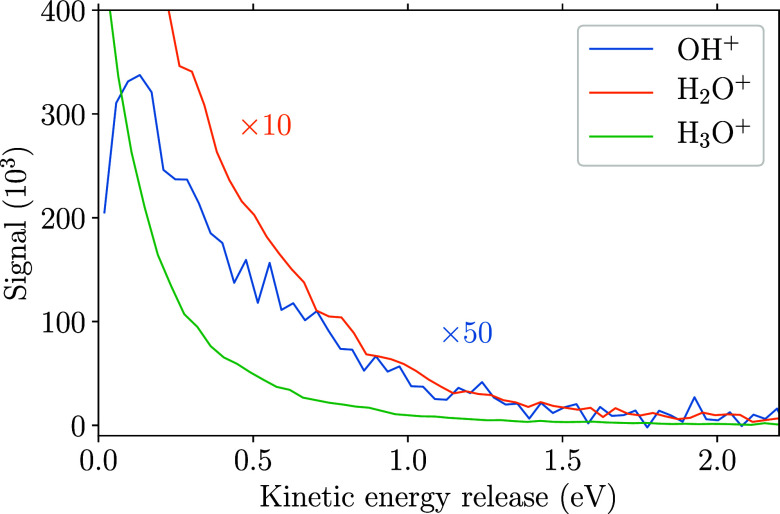
Kinetic-energy release of the reaction channels yielding the specified ions, assuming two-body fragmentation.

Besides the mass spectrometric information about the created ions and their signal, in VMI, we also obtain insights into the energy redistribution for the specific fragmentation channels through the distributions of kinetic-energy releases, which are displayed for some selected ions in Figure 3; for comparison, see also the released-total-momentum distributions in Figure S3 of the Supporting Information. The kinetic-energy-release spectra were obtained from the measured 3D single-ion momenta, assuming two-body fragmentation of the parent ion.

This is a valid assumption for the H_3_O^+^ ions and the larger part of the H_2_O^+^ signal according to channels (1) and (3), respectively, with the calculated kinetic-energy release equal to the total kinetic energy *E*_tr_. However, the OH^+^ ions were produced from three-body fragmentation according to channel (5). Here, the most probable scenario is a sequential fragmentation into OH^+^ with an intermediate H_3_O, which further dissociates into H_2_O and H. Thus, the plotted curve of the kinetic-energy release in the OH^+^ reaction channel illustrates the kinetic energy released in the first step of the three-body fragmentation.

The low-energy peaks up to 2 eV in the *E*_tr_ spectra are attributed to the decay of various rovibrational states of (H_2_O)_2_^+^. The individual curve shape then results from the convolution of initial rovibronic excitation of (H_2_O)_2_^+^ and subsequent statistical fragmentation. From these distributions, rovibronic excitation energies *E*_rv_ can be obtained by assuming the conservation of energy given by *E*_total_ = *E*_tr_ + *E*_rv_. In a state-selective experimental approach, such a constraint would allow for the calculation of the exact rovibronic excitation of the generated fragments. However, limitations arise in the ultrashort-pulse ionization regime due to the spectral bandwidth of the ionizing laser, ∼30 nm in our case. Assuming that the deposited energy during the (H_2_O)_2_ ionization is given by the lowest number of photons necessary to overcome the energy threshold, i.e., the appearance energy *E*_A_, of the selected fragmentation-reaction channel and considering the effective spectral narrowing due to the multiphoton ionization, one could extract low-resolution (>0.04 eV) rovibrational spectra using the dissociation threshold for each detected fragment.

Table 1 provides a summary of previously reported and our new findings: A list of all detected ions from decay of (H_2_O)_2_^+^ is shown in the first column. Seven of the observed ions can be linked to the already reported pathways from simulations.^15,16^ The additionally detected ions are created through previously unreported fragmentation channels. The fragmentation pathways leading to the ejection of the newly discovered ions and those discussed in the introduction are shown in the last column. These suggested pathways are based solely on energetic arguments and thus should be further investigated, e.g., by computations or through radical detection. From our data, we cannot directly link the detected ions to the exact initial state of the ion. Nevertheless, we can count the number of detected ions and determine their relative ion yields, which are given in the second and third columns, respectively.

**Table 1:** Summary of All the Observed Ions from (H_2_O)_2_^+^ Dissociation Together With Their Relative Ion Yields^a^

fragment	signal (counts)	relative ion yield	*E*_A_ (eV)	reaction channel:
				(H_2_O)_2_^+^ →
H_3_O^+^	1,933,751	0.541(42)	11.7^15^	H_3_O^+^ + OH^15,16,19^
H_2_O^+^	630,492	0.176(11)	12.8^15^	H_2_O^+^ + H_2_O^15,16,19^
			18.2^15^	H_2_O^+^ + OH + H^15^
(H_2_O)_2_^+^	613,728	0.172(8)	11.7^15^	H_3_O^+^···OH^15,16,19^
H^+^	213,905^b^	0.06(4)	19.2^15^	H^+^ + H_2_O + OH^15^
OH^+^	114,390	0.032(8)	18.5^15^	OH^+^ + H_2_O + H^15^
O^+^	24,235	0.0068(20)	**21.7**	O^+^ + H_2_O + 2H
H_2_^+^	13,947	0.0068(14)	**21.7**	H_2_^+^ + H_2_O + O
H_2_^18^O^+^	10,225	0.0029(1)		
H_3_^18^O^+^	9268	0.0026(3)		
O_2_^+^	6052	0.0017(3)	**27.6**	O_2_^+^ + 4H
HO_2_^+^	4206	0.0012(2)	**29.2**	HO_2_^+^ + 3H
(H_2_O–O)^+^	1792	0.0005(1)	32.6^16^/**33.0**	(H_2_O–O)^+^ + 2H^16^
H_3_^+^	796	0.0002(3)	**36.5**	H_3_^+^ + HO_2_

a Our estimated appearance energies *E*_A_ are shown in the fourth column in bold, alongside the previously reported appearance energies.^15,16^ The last column shows previously reported and suggested fragmentation pathways producing the observed ions.

b Signal contributions by fast nondetected H^+^ ions are expected to be below 5% of the integrated H^+^ signal.

**Figure 4:**
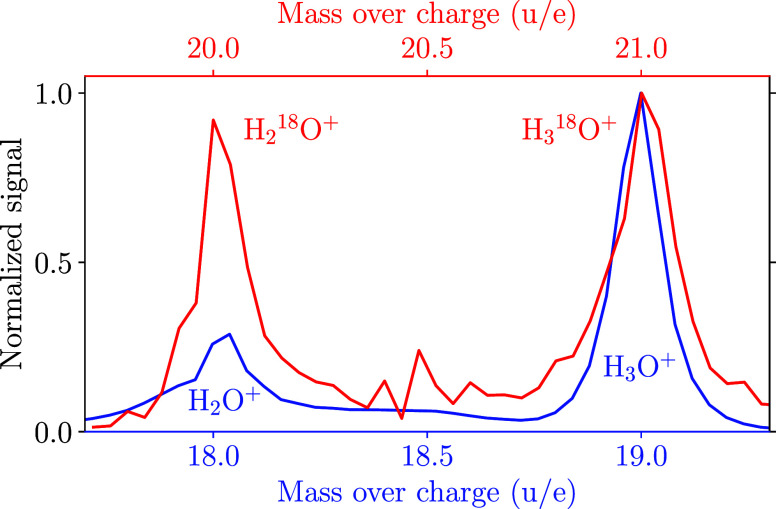
Comparison of the peak-normalized mass spectra including the signals assigned to (blue) H_2_O^+^ and H_3_O^+^ and (red) H_2_^18^O^+^ and H_3_^18^O^+^. The red line is background-corrected to exclude the background of the H_2_^18^O^+^ peak centered at 20 u/e. This is shown in Figure S6 in the Supporting Information. See also the text for details on possible small ^17^O backgrounds.

Upon inspecting the ion yields, an intriguing observation is that while the signal intensity of H_3_O^+^ is roughly 3 times larger than the signal of H_2_O^+^, the situation is changed for their isotopologues H_3_^18^O^+^ and H_2_^18^O^+^, which exhibit comparable signal intensities, see Figure 4. With the natural abundance of ^17^O and the sensitivity of our experiment, one would also expect to observe H_3_^17^O^+^ and H_2_^17^O^+^. Unfortunately, these signals are hidden under the stronger signals of H_2_^18^O^+^ and H_3_O^+^, respectively. For the H_3_O^+^ signal, a background contribution from H_3_^17^O^+^ is below 0.04% and thus negligible. For the H_2_^18^O^+^ peak at *m*/*q* = 20 u/e, a contribution by H_3_^17^O^+^ could be as large as 20%. In any case, the difference in the ratios for the H_2,3_^16^O and H_2,3_^18^O signals is a surprisingly strong illustration of isotopic substitution modifying chemical-reaction pathways. This could be due to a preferred stereometric position of H_2_^18^O as a proton donor in (H_2_O)_2_, or it could be due to a reduced proton tunneling and transfer probability between the two water moieties in the ^18^O isotopologue, which could both be a consequence of small reduced-mass changes resulting in zero-point-energy and anharmonic-coupling effects.

On top of the qualitative analysis, the obtained relative ion yields were also utilized to estimate the appearance energies *E*_A_ of the newly detected ions and assigned channels. Within our model, the relative ion yields can be expressed as
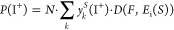
8*N* is a normalization factor and *y*_*k*_^*S*^ is the branching ratio of the channel *k* producing ion I^+^ after ionization into an initial ionic state *S*. The probability to ionize into such a state *S* is described by the distribution *D* as a function of the ionization energy *E*_i_ of the state *S* and a parameter *F* corresponding to the strength of the applied external electric field.^45^ The *D* was chosen to have an exponential character based on the strong-field ionization approximation.^45^ The unknown parameters of our model, i.e., *F* and the branching ratios of H^+^ and OH^+^ ions, were calibrated using the theoretical branching ratios and *E*_A_ of each channel given by Svoboda et al.^15^ We then calculated the so far unknown *E*_A_ of the less abundant ions from (8), see the Supporting Information for further details. These estimated *E*_A_ values are shown in Table 1 including the reported ones.^15,16^ This includes the *E*_A_ = 33.0 eV of (H_2_O–O)^+^ known from reference (16) to be 32.6 eV. The surprisingly good agreement should be taken with caution and not as an illustration of the high precision of the presented ad hoc model. The calculated and reported appearance energies *E*_A_ assigned to the observed ions directly reflect the broad range of the initial energies deposited by the strong-field photoionization triggering the (H_2_O)_2_^+^-fragmentation reactions.

## Conclusions

Overall, our study provides unique novel experimental observations of the (H_2_O)_2_^+^-fragmentation pathways, which were previously only, and only partly, predicted by molecular dynamics simulations.^15,16^ These results substantially broaden our perspective on (H_2_O)_2_^+^ fragmentation by showing an additional set of newly observed ion-radical pathways and their relative ion yields. Together with our estimated appearance energies, *E*_A_, these indicate the relative significance among all detected channels.

The observed very strong O-isotope effect indicates nonclassical aspects of molecular structure and reactivity in the fragmentation. Together with the experimental information on kinetic energies and angular distributions of all fragments, these data are a very valuable asset for advanced molecular dynamics simulations unraveling the underlying chemistry. Time-resolved investigations of the ionization and subsequent reaction pathways of water dimer could provide further insights into its electronic and nuclear dynamics, including the time scales of proton and hydrogen transfer, the traversal of electronic states, and electronic relaxation processes.

Our findings are relevant for discussions of the role of water ionization and the produced ionic and radical fragments as triggers of the subsequent ion-radical photochemistry, e.g., on ice mantels of dust particles in interstellar space. They also generally help to disentangle a broad inventory of environmentally important radicals.

## Data Availability

The scripts used to analyze the recorded data and the specified equations are available from the corresponding author upon request.

## Supporting Information Available

Experimental ion-imaging map with the deflector-electrodes grounded; photoion–photoion coincidence map; description of a model for calculating the appearance energies; plot of the total-momentum release for specified ions; dependence of the appearance energy on the relative ion yields; and details on the correction of the background in the calculations of the H_2_^18^O^+^/H_3_^18^O^+^ ratio (PDF)
