# Supplementary material for: Reaction Pathways of Water Dimer Following Single Ionization
Source: J Phys Chem A. 2024 Feb 26;128(9):1593–9. doi: 10.1021/acs.jpca.3c07958 (PMC10926096; doi:10.1021/acs.jpca.3c07958)
Supplement: Supplementary file 1 — jp3c07958_si_001.pdf [file jp3c07958_si_001.pdf]

# *Supporting Information for Publication:* Reaction Pathways of Water Dimer Following Single Ionization

Ivo S. Vinklárek,[1] Hubertus Bromberger,[1] Nidin Vadassery,[1]
Wuwei Jin,[1] Jochen Küpper,[1,2,3,*] and Sebastian Trippel[1,2,†]

[1] *Center for Free-Electron Laser Science CFEL, Deutsches Elektronen-Synchrotron DESY, Notkestr. 85, 22607 Hamburg, Germany*

[2] *Center for Ultrafast Imaging, Universität Hamburg, Luruper Chaussee 149, 22761 Hamburg, Germany*

[3] *Department of Physics, Universität Hamburg, Luruper Chaussee 149, 22761 Hamburg, Germany*

(Dated: 2024-01-18)

## CONTENTS

List of Figures S1

List of Tables S1

Model S2

Experimental results S3

Ratio of $H_2{}^{18}O^+/H_3{}^{18}O^+$ S3

References S4

## LIST OF FIGURES

S1 Map of the ion signal without deflection S2

S2 Photoion-photoion correlation . . . . . . . . . S2

S3 Total-momentum releases . . . . . . . . . . . . . S3

S4 Appearance energies . . . . . . . . . . . . . . . . . S3

S5 Isotopologue intensities . . . . . . . . . . . . . . S4

S6 Background correction . . . . . . . . . . . . . . . S4

## LIST OF TABLES

S1 Branching ratios . . . . . . . . . . . . . . . . . . . . S2

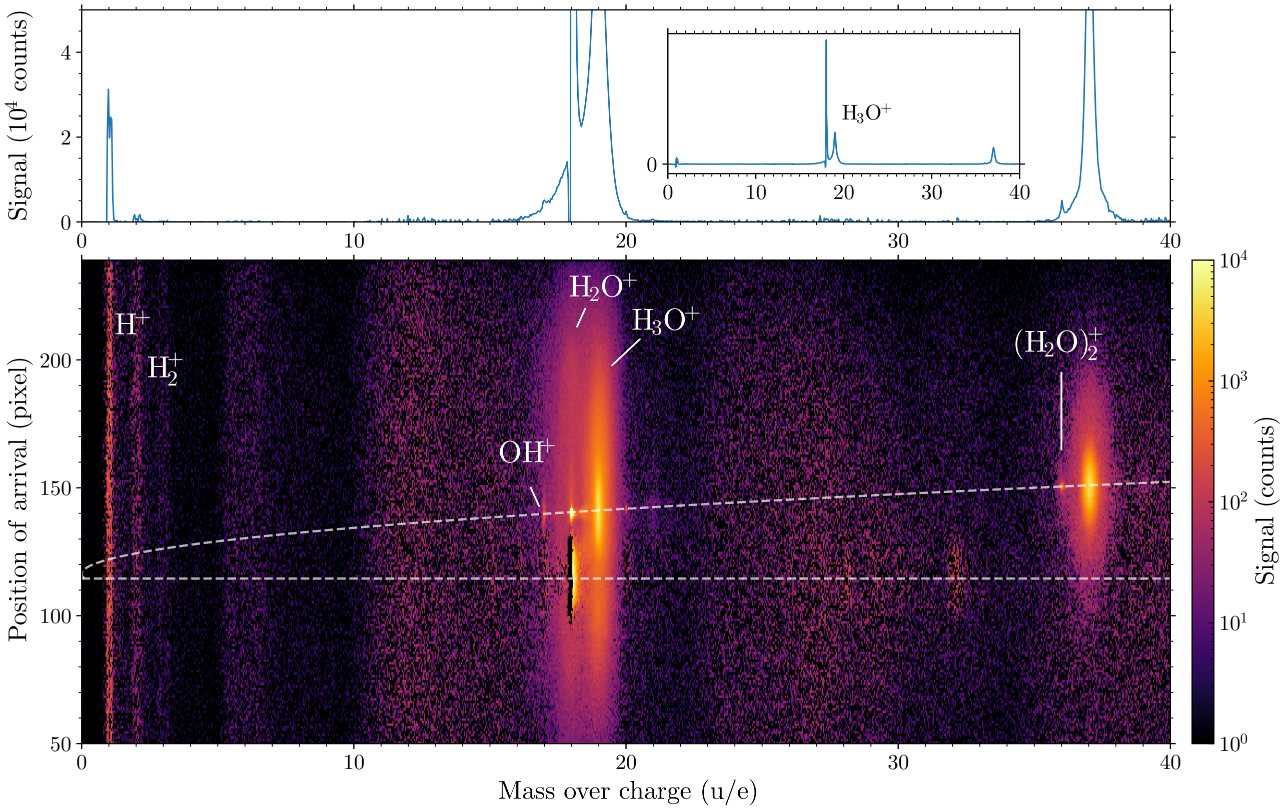


Figure S1. Image mapping the ion signal according to the mass-over-charge ratio and the position of arrival at the detector. This two-dimensional spectrum was obtained with deflector off.

## MODEL

To estimate the appearance energies for ions with low abundances, i. e., $O_2^+$, $HO_2^+$, $(H_2O\text{-}O)^+$, $H_2^+$, and $H_3^+$, we designed a semi-empirical model combining the measured relative ion yields with reported ionization energies $E_\mathrm{i}$ and branching ratios [1]. For the calculation of the ion yield of $I^+$ we applied the equation

$$P(\mathrm{I}^+) = N \cdot \sum_k y_k^S(\mathrm{I}^+) \cdot D(F, E_\mathrm{i}(S)), \tag{1}$$

where $N$ is a normalization factor, $y_k^S$ is the branching ratio of the channel $k$ after ionization into state $S$ and $D$ is a distribution describing the ionization probability. $D$ depends on the parameter $F$ corresponding to the strength of the applied external field [2] and the ionization energy $E_\mathrm{i}(S)$ of state $S$. (1) was rewritten into the following equations using the reported

TABLE S1. Comparison of the measured and calculated branching ratios for the selected ions.

| Ion | $H_3O^+$ | $H_2O^+$ | $(H_2O)_2^+$ | $H^+$ | $OH^+$ |
|---|---|---|---|---|---|
| Measured | 0.541 | 0.176 | 0.172 | 0.060 | 0.032 |
| Calculated | 0.494 | 0.180 | 0.233 | 0.061 | 0.033 |

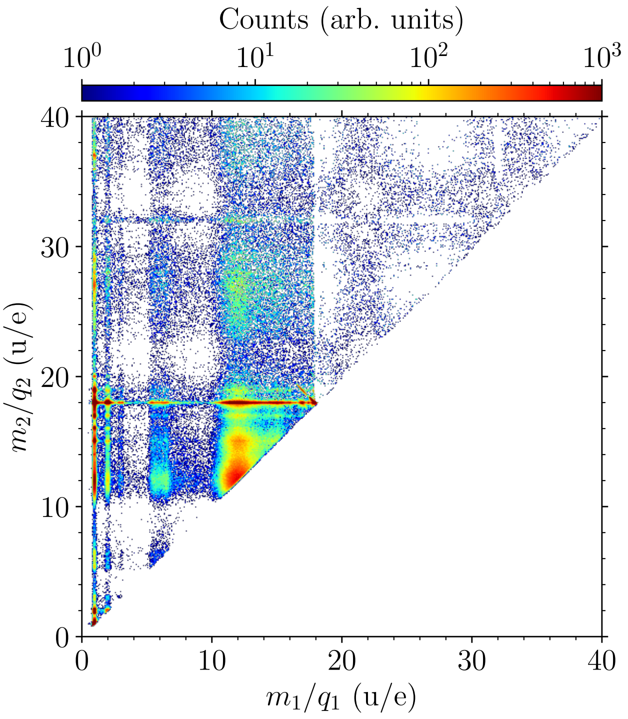


Figure S2. Photoion-photoion correlation for the mass-over-charge range between 0 and 40.

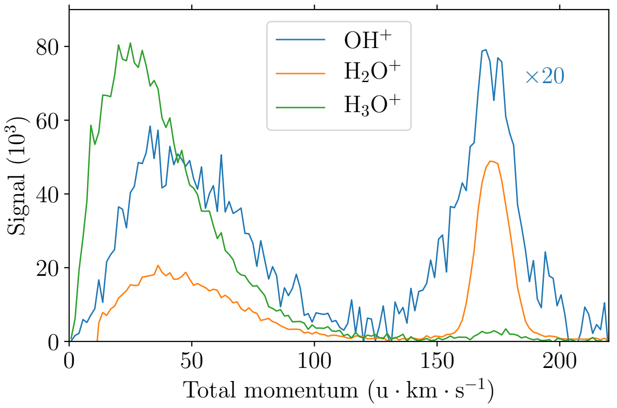


Figure S3. Total-momentum release of the reaction channels yielding the specified ions with the assumption of two-body fragmentation.

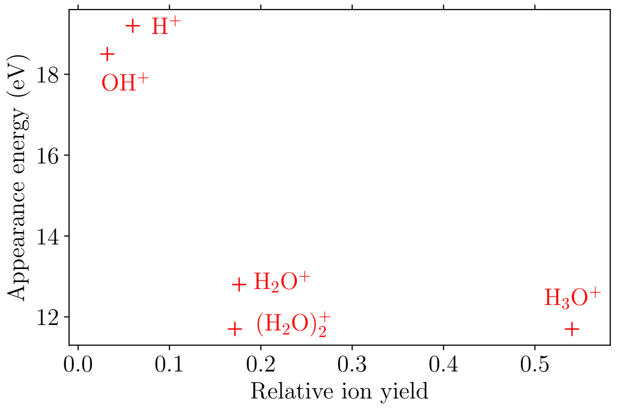


Figure S4. Dependence of the appearance energy $E_\mathrm{A}$ on the relative ion yields for $H_3O^+$, $H_2O^+$, $(H_2O)_2^+$, $OH^+$, and $H^+$ ions.

parameters [1]

$$P(H_3O^+) = N \cdot (D(F, 11.7) \cdot 0.635 + D(F, 12.8) \cdot 0.66 + D(F, 18.2) \cdot 0.14), \tag{2}$$

$$P((H_2O)_2^+) = N \cdot (D(F, 11.7) \cdot 0.365 + D(F, 12.8) \cdot 0.245), \tag{3}$$

$$P(H_2O^+) = N \cdot (D(F, 12.8) \cdot 0.095 + D(F, 18.2) \cdot 0.86 + D(F, 18.5) \cdot (1 - p_{OH^+}) + D(F, 19.2) \cdot (1 - p_{H^+})), \tag{4}$$

$$P(OH^+) = N \cdot (D(F, 18.5) \cdot p_{OH^+}), \tag{5}$$

$$P(H^+) = N \cdot (D(F, 19.2) \cdot p_{H^+}). \tag{6}$$

The parameters $F$, $p_{OH^+}$, and $p_{H^+}$ were optimized to fit the measured and calculated relative ion yields. Assuming $D(F, E_\mathrm{i}) = \exp(-E_\mathrm{i}/F)$, this yielded $F = 4.34$ eV, $p_{OH^+} = 0.37$ and $p_{H^+} = 0.82$. The calculated and measured relative ion yields are shown in the Table S1. The fitted field-strength parameter $F$ was then applied to estimate appearance energies $E_\mathrm{A}$ of other ions $I^+$ with low abundance according to

$$E_\mathrm{A}^{I^+} = -F \cdot \log(P(I^+)) \tag{7}$$

where we set branching ratio equal to 1. The evaluated appearance energies are shown in the fourth column of Table 1 in the main text.

Finally, we would like to point out that the fitted value of $F = 4.34$ eV corresponding to the strength of the applied external field agrees well with the ponderomotive energy $U_\mathrm{p}$ multiplied by 1/e, i. e.,

$$U_\mathrm{p} = \frac{E_\mathrm{i}}{2\gamma^2} \cdot \frac{1}{\mathrm{e}} = 4.39 \text{ eV}, \tag{8}$$

$E_\mathrm{i}$ is the ionization energy of water dimer (11.7 eV) and $\gamma = 0.7$ the corresponding Keldysh parameter. Thus, it is tempting to assign a real physical meaning to the parameter $F$ as the "averaged" ponderomotive energy. Nevertheless, further modelling of our experiment is necessary to confirm this hypothesis.

## EXPERIMENTAL RESULTS

Here, we provide additional information on the experimental results discussed in the main text. Figure S1 displays the acquired background-subtracted ion signal with the deflector off ($U_\mathrm{d} = 0$ kV). The acquired signal illustrates the direct-molecular-beam background of our measurement. Figure S2 shows results of our photoion-photoion covariance analysis for the mass-over-charge range between 0 and 40. The fact that there are no cross-correlation peaks for the range 32–34 u/e support the origin of the $O_2^+$, $HO_2^+$ and $(H_2O\text{-}O)^+$ ions in the $(H_2O)_2^+$ fragmentation.

Figure S3 shows the total-momentum release for the specified ions following strong-field ionization of $(H_2O)_2$. Figure S4 shows the correlation between the reported appearance energies $E_\mathrm{A}$ and the measured relative ion yields.

## RATIO OF $H_2{}^{18}O^+/H_3{}^{18}O^+$

The ion count ratios of $H_2O^+/H_3O^+ \approx 3$ and $H_2{}^{18}O^+/H_3{}^{18}O^+ \approx 1$, discussed in the main text, are determined from the signals plotted in Figure 1 in the main text. The ratios indicate different dynamics for the respective $^{18}O$ and $^{16}O$ isotopologues. Figure S5 shows the ion-imaging maps in the areas of the $H_2{}^{18}O^+$ and $H_3{}^{18}O^+$ signals together with the region of interests used to obtain the ion counts, i. e., 10225 and 9225, respectively. This was further supported by the areas in the mass spectrum corrected for the signal

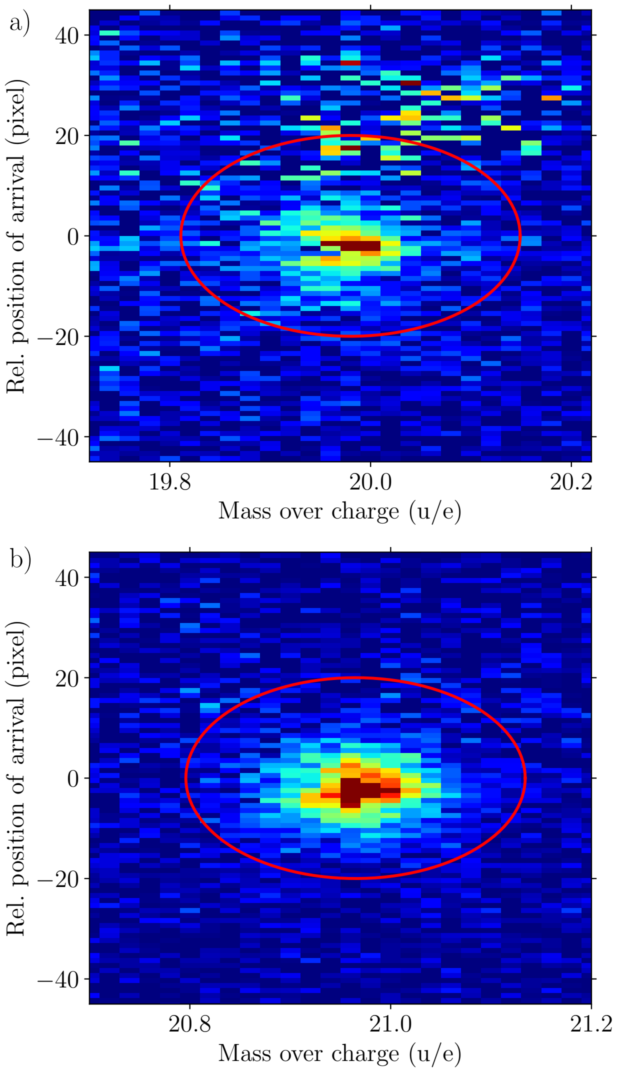


Figure S5. Ion-imaging maps for (a) $H_2{}^{18}O^+$ and (b) $H_3{}^{18}O^+$ signals with the regions of interest used for the ion counting marked by red ellipses.

due to the broad distribution of the $H_3O^+$ peak, see Figure S6.

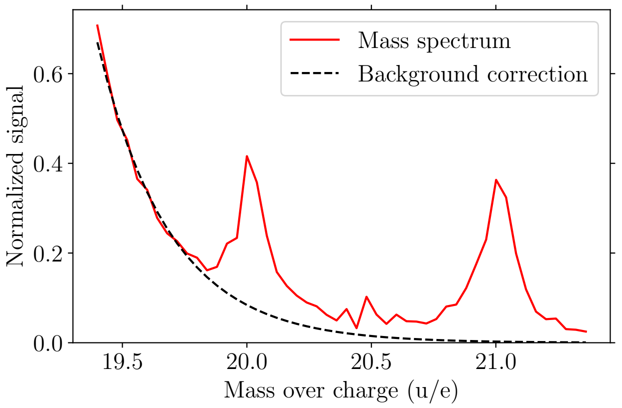


Figure S6. Mass spectrum in the $^{18}$O-isotopologue range with the fitted background from $^{16}$O isotopologues.

* Email: jochen.kuepper@cfel.de
† Email: sebastian.trippel@cfel.de;
Website: https://www.controlled-molecule-imaging.org

[1] O. Svoboda, D. Hollas, M. Ončák, and P. Slavíček, Reaction selectivity in an ionized water dimer: Nonadiabatic ab initio dynamics simulations, Phys. Chem. Chem. Phys. **15**, 11531 (2013).

[2] M. V. Ammosov, N. B. Delone, and V. P. Krainov, Tunnel ionization of complex atoms and of atomic ions in an alternating electromagnetic field, Sov. Phys. JETP **64**, 1191 (1986).
